# Brain Damage Treated with Non Proven Intensive Training 2003-2011: A Norwegian Cost Analysis

**DOI:** 10.5539/gjhs.v4n6p179

**Published:** 2012-10-10

**Authors:** Jan Norum, Arnborg Ramsvik, Knut Tjeldnes

**Affiliations:** 1 Northern Norway Regional Health Authority trust, Bodø, Norway; 2 Department of Oncology, University Hospital of North Norway, Tromsø, Norway; 3 Institute of Clinical Medicine, Faculty of Health Sciences, University of Tromsø, Tromsø, Norway

**Keywords:** brain damage, cost, training, non-proven, Norway

## Abstract

**Objectives:**

There has been an increased request for intensive training and rehabilitation of patients with brain damage in Norway. These programs are demanding with regard to personnel, travelling, time and economic resources. We aimed to indicate cost and gain to make these programs cost-effective.

**Methods:**

A retrospective study included all patients referred to the Northern Norway Regional Health Authority (NNRHA) trust during the nine years period 2003-2011. All referrals to the NNRHA trust for the economic coverage of foreign based rehabilitation or habilitation programs (The Advanced Bio-Mechanical Rehabilitation (ABR), Institutes of Achievement of Human Potential program (IAHP) (Doman method), Family Hope Center (FHC) program and the Kozijavkin method) were included. 17 patients were detected and 15 fulfilled the inclusion criteria for funding. Median age was 8 years (1-31 years). Cost from the specialist health care point of view was calculated. A cut-off limit of €57,000/quality adjusted life year (QALY) and a 4% discount rate was employed.

**Results:**

The undiscounted cost per patient enrolled was calculated €133,210 (discounted €121,348). To make these therapies cost effective, a total of at least 2.13 QALYs (2.34 undiscounted QALYs) must be gained per patient enrolled. Such a gain could not be indicated and we doubt it is achievable.

**Conclusion:**

Non-proven intensive training programs for patients with brain damage are costly. As long as their effect has not been documented, health care services should not spend resources on these programs outside clinical trials.

## 1. Introduction

During the last decade, there has been an increased request of intensive training and rehabilitation programs for patients with brain damage (Norwegian Board of Health Supervision, 2000). Based on a political decision, parents of children with brain damage may receive economic coverage from the Regional Health Authority (RHA) trusts for intensive training programs based outside Norway (Ministry of Health and Care Services, 2004). The programs available for coverage were the Advanced Bio-Mechanical Rehabilitation (ABR), Institutes of Achievement of Human Potential program (IAHP) (Doman method), Family Hope Center (FHC) program and the Kozijavkin method. These programs are demanding with regard to personnel, travelling, time and economic resources. Despite families’ requests for these therapies during many years and economic coverage of treatment, randomized clinical trials have been absent. The Norwegian Knowledge Centre for the Health Services (NKCHS, 2008) searched systematically international databases for publications in this field. They did not find any systematic reviews or controlled clinical trials that evaluated the effect of foreign programs as ABR, IAHP/Doman, FHC program or the method of Kozijavkin. Consequently, they concluded we do not know the effect of these programs.

In northern Norway several clinicians have criticized the economic resources spent on these non-proven therapies and argued for an allocation of these resources to national rehabilitations programs. We therefore decided to analyze the resources spent on the foreign rehabilitation/habilitation programs and estimate the necessary quality of life gained to make these investments cost-effective.

## 2. Methods

### 2.1 Study Design/Settings

In February and March 2012, all requests to the Northern Norway Regional Health Authority (NNRHA) trust for economic coverage of foreign based rehabilitation or habilitation programs during the nine years time period 2003-2011 were retrospectively analyzed.

According to Norwegian policy, patients or their parents may request for economic coverage of intensive rehabilitation/habilitation programs for the treatment of congenital or acquired brain injury. The criteria employed were: The intensive programs may range from training three times per week to several times per day. They must be focused and aim for an improved quality of life by enhancing the patient’s mobility, communication or social and/or mental functioning. The various programs are summarized in Table 1. The economic support was approved for one year and a new request was necessary for continued coverage.

**Table 1:** A short overview of foreign based rehabilitation/habilitation programs financed by the Norwegian health services

*Advanced Bio-Mechanical Rehabilitation (ABR)*	The method was established in 1995 and got its present name in 2002. The treatment is offered in many countries by the ABR International. The treatment has a bio-mechanic approach. The treatment aims to strengthen the smooth muscles of the internal organs (“the hydraulic skeletal”). The patients are treated 20 hours a week by their parents and the family is offered guidance and supervision four times a year within one of the ABR centers (www.blyum.com).

*Institutes of Achievement of Human Potential program (IAHP/Doman method)*	It was developed in Philadelphia by Glenn Doman in 1955. The treatment focus on the brain and aims to stimulate healthy parts to take over the functions lost in the damaged areas. According to the program, educated parents have to stimulate their children 8-12 hours daily and visit the institute in USA twice a year for evaluation and guidance (www.iahp.org).

*Family Hope Center program*	The FHC is also located in Philadelphia USA. Their program has a similar focus as the IAHP method, but offers more freedom. Consequently the combination of treatment, normal family life and kindergarten is more achievable (www.familyhopecenter.org).

*Kozijavkin Method*	Offered by the International Clinic of Rehabilitation (ICR) in Ukraine. It was developed in 1980 and focus on biomechanical corrections of the joints combined with mobilizing exercises, reflex-therapy, various massages, acupressure and mechano-therapy. The treatment is initiated with 2 weeks intensive therapy at the ICR, followed by 6-8 months home based therapy and visits to the ICR twice a year (www.rehab.lviv.ua)

### 2.2 Material

A total of 55 requests (17 patients) for economic support were registered during study period. Fifteen patients fulfilled the inclusion criteria for economic support and they were included in the survey. Patient characteristics are shown in Table 2 and their treatment programs in Table 3. Cost was calculated according to data from the NNRHA trust and converted into Euros (€) at a rate of 1 Euro = 7.4225 Norwegian Krone (NOK), as of 1^st^ of March 2012 (www.norges-bank.no). The suggested Norwegian cut-off limit with regard to cost per quality adjusted life year (QALY) gained has been suggested €57.000 (The Norwegian handbook of medication group, 2010). This figure was implemented in the analysis.

**Table 2:** The table shows patient characteristics

Characteristics		Number	Median	Range
Total		15 patients		
Gender	Females	8 patients		
	Males	7 patients		
Age			8 years	1-31 years
County	Finnmark	6 patients		
	Troms	4 patients		
	Nordland	4 patients		
	Not registered	1 patients		

**Table 3:** The table shows treatments employed

Treatment method	Numbers
Advanced Bio-Mechanical Rehabilitation (ABR)	1 patients
Institutes of Achievement of Human Potential program (IAHP)/Doman	11 patients
Family Hope program	3 patients
Method of Kozijavkin	0 patients

### 2.3 Statistical Analysis, Discount Rate and Authorisation

The Microsoft Excel 2007 version was employed for the final database and calculations. The study was performed as an economic “quality of care analysis” and consequently no approval from the Regional Committee for Medical and Health Research Ethics (REK) was necessary. Similarly, no approval from the Norwegian Social Science Data Services (NSD) was requested. A 4% discount rate (d.r.) was employed.

## 3. Results

**Figure 1:**
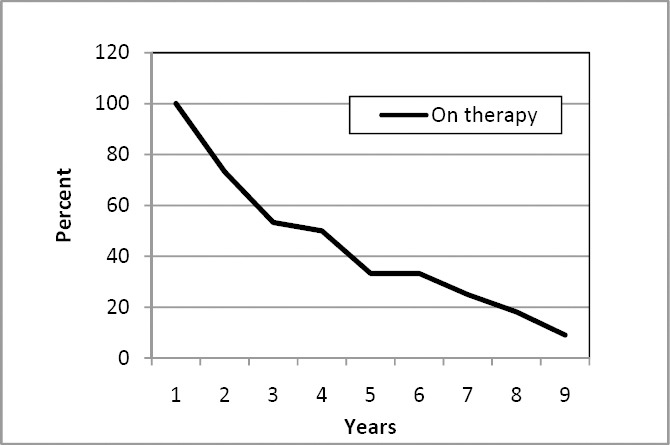
The figure shows the percentage of patients in foreign based rehabilitation programs according to years after initiation. The Kaplan Meier method was employed

The number of patients included in the foreign based non-proven rehabilitation/habilitation programs dropped during follow up. Two years after initiation of therapy, one fourth had left the program. At four years follow up, half of patients had ended therapy. Further details are illustrated by the Kaplan-Meier curve shown in Figure 1.

During study period a total of €1.76 million (treatment €1.63 million and equipment €0.13 million) was spent on therapy, leaving a total figure of €117,515 per patient enrolled. Employing the Kaplan-Meier method (Figure 1), the undiscounted cost per patient enrolled was €133,210 (discounted €121,348).

**Figure 2:**
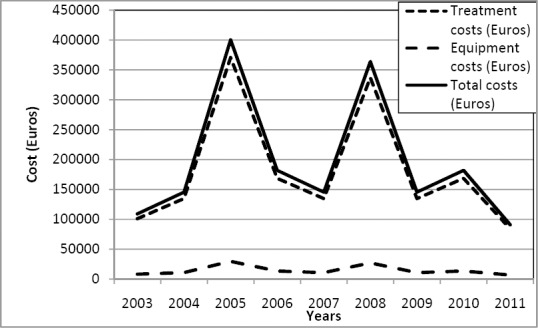
TThe figure shows the annual cost of foreign based non-proven rehabilitation. The costs (in Euro) are shown according to treatment, equipment and total costs

The median age was eight years. Life expectancy among children with cerebral palsy has been published in a prior study (Strauss et al., 2008). The figures at age 15 years varied significantly between various levels of functioning (13-55 years). Based on this study, we assumed patients in our survey (median age 8 years) would have a mean life expectancy of 45 years. Employing this figure and the suggested Norwegian cut-off limit (€57,000/QALY) and a 4% d.r., the annual gain in quality of life was calculated. To make this therapy cost effective, a mean total gain of at least 2.13 QALYs (2.34 undiscounted QALYs) must be achieved per patient enrolled. Assuming these programs only improves quality of life (no survival gain), the annual improvement must be at least 0.1 (undiscounted 0.05).

## 4. Discussion

Despite the fact that these patients were young (median age 8 years) and could expect many life years, the numbers on therapy dropped significantly during follow up. Within four years, half of patients dropped out of the treatment plan. This strongly indicated that the intensive training programs are exhausting both for the children as well as their parents. The dropouts may also signalize that most parents did not find the gain achieved (if any) worth all its efforts. Consequently, they ended their participation in the treatment plan.

To make this therapy cost effective a total of 2.13 quality adjusted life years (QALYs) (2.34 undiscounted QALYs) had to be gained per patient enrolled. We employed the results of Strauss et al. (2008) and estimated roughly a life expectancy of 53 years in this group of patients. However, the survival of patients with cerebral palsy varies significantly depending on severity (Strauss et al., 2007; Strauss, 2010). Those fed by others and who did not lift their heads in prone or roll or sit independently had at 4 years of age a life expectancy of only 11 years. However, a significant improvement was observed during the last 20 years. Life expectancy among this group was calculated 20 years in 2007. Consequently, life expectancy varies significantly depending on level of functioning and seems to be improving (Strauss et al., 2007; Brooks et al., 2007). Obviously, we employed a rough estimate. As long as it cannot be documented that intensive training programs improves survival, only quality of life can be considered in health economic analyses. Furthermore, quality of life varies during life and consequently possible QALYs gained may vary between individuals and year of life. Our cut-off limit was a significant improvement in QoL Can such a gain be achieved? Pietrzak (2001) concluded the theoretical foundations and clinical efficacy of the IAHP rehabilitation techniques had not yet been adequately documented. Hadders-Algra and colleagues (2005) published in Lancet that they were not aware of any evidence of the effectiveness of the Kozijavkin method. Furthermore, they argued that the method gave parents false hope. Based on this information and the fact that no documented quality of life improvement was revealed by the Norwegian Board of Health Supervision (2000), we doubt that this treatment can be cost-effective. On the other hand, The Institutes for the Achievement of Human Potential published a retrospective single arm study including a small series of 21 children with cortical visual impairment who had undergone an intensive visual stimulation program. They concluded their results indicated (even in their challenging group) a considerable neuroplasticity in visual systems leading to reintegration and visual recovery (Malcowicz et al., 2006). However, any proof of effectiveness cannot be obtained from a non-randomized study.

It could be argued that a full health technology assessment (HTA) is necessary to clarify the cost-effectiveness of these programs. However, a cost minimizing method is a suitable alternative when other costs (than those analyzed) are equal in both arms. In this study, the alternative was a none specialized health care (SHC) service. However, we cannot exclude the possibility that some activities may have been offered from the SHC-services in a suggested “none intervention” setting. However, the possible costs of such activities may be balanced by the cost of support from the primary health care and the local community in an intervention arm. Furthermore, neither the total cost of possible alternatives nor the gain (QALYs or LYs) achieved by the intensive training programs can be indicated. Consequently, no HTA can be performed.

Whereas there was no literature on cost or effectiveness with regard to these training programs, it does not mean that there is no literature at all. We searched in September 2012 the PubMed database (www.ncbi.nlm.nih.gov/pubmed) employing the following search criteria: Kozijavkin (2), Advanced Biomechanical Rehabilitation (51) and Doman method (46). The numbers in parenthesis are the number of hits. Dr. Doman and colleagues published in 1960 (Doman et al., 1960) beneficial results of their treatment, but neither a control group nor standardized statistical procedures were used. Holm (1983) published an overview of the Doman-Delacato treatment. They reviewed several publications by Delacato and Doman and concluded that these studies do not stand up to scientific scrutiny. Two well-designed studies (Neman et al., 1975; Sparrow et al., 1978) used two control groups. Whereas the Neman study showed no dramatic cases of improvement and no changes in global intelligence, some minimal positive effects on tests of visual perception and language were revealed. The work of Sparrow and colleague (1978) showed no difference in posttest performance effects among any of the three groups. Furthermore, harmful effects on burdened and confused parents given false hope and increasing guilt were reported (Holm 1983). Especially, the rigid and inflexible time schedule of the IAHP (12 hours a day, seven days a week) has been demanding (Freeman, 1967).

The Kozijavkin method was described in Lancet a few years ago by Ben Aris (2005). Their presentation was later commented by Hadders-Algra et al. (2005) who argued that Aris’ account might give many parents false hope. They claimed there is no evidence of the effectiveness of the Kozijavkin method and advised the Ukrainian colleagues to report their results in a peer-reviewed journal. The lack of documentation may have been noted by Norwegian parents as none requested this therapy in our survey.

With a low number of patients and consequently less cost, health care administrators have been more willing to allocate resources to new therapies and spend more than the recommended cut off limit among such groups. In Norway (5 million inhabitants), there are annually between 2,000 and 2,500 children born (total annual number of births about 62,000) with any form of reduced functioning. Furthermore, children injured due to accidents or serious chronic diseases may experience reduced functioning. In total, 2-2.5% of these children will be in need of rehabilitation/habilitation services (Andersen et al., 2007). The frequency of cerebral palsy (CP) is about 2.1 per 1,000 births and consequently there are about 120 new cases each year (3). The frequency of myelomeningocele is 0.5/1,000 births and the numbers of serious acquired brain injury has been reported 10-15 cases/year among children below the age of 15. In total there have been reported 30-35 children with serious acquired brain injury and in need of rehabilitation services annually (Norwegian knowledge centre, 2008). Knowing northern Norway has 9% of the Norwegian population, the annual figure within the region can be indicated 3 children per year. Based on this assumption and the fact that there may be a pool of patients when the coverage was introduced, it can be indicated that less than half of candidates for intensive therapy asked for funding.

## 5. Conclusion

In conclusion non-proven intensive training programs for patients with brain damage was costly. To make this therapy cost effective, a significant gain in quality of life and/or life years gained must be achieved. As long as this gain cannot be indicated or documented, health care services should not spend resources on these programs outside randomized clinical trials.
